# Current (Food) Allergenic Risk Assessment: Is It Fit for Novel Foods? Status Quo and Identification of Gaps

**DOI:** 10.1002/mnfr.201700278

**Published:** 2017-12-11

**Authors:** Gabriel Mazzucchelli, Thomas Holzhauser, Tanja Cirkovic Velickovic, Araceli Diaz‐Perales, Elena Molina, Paola Roncada, Pedro Rodrigues, Kitty Verhoeckx, Karin Hoffmann‐Sommergruber

**Affiliations:** ^1^ Laboratory of Mass Spectrometry – MolSys Department of Chemistry University of Liege Liege Belgium; ^2^ Division of Allergology Paul‐Ehrlich‐Institut Langen Germany; ^3^ Center of Excellence for Molecular Food Sciences University of Belgrade – Faculty of Chemistry Belgrade Serbia; ^4^ Ghent University Global Campus Yeonsu‐gu Incheon South Korea; ^5^ Center for Plant Biotechnology and Genomic (UPM‐INIA) Madrid Spain; ^6^ CIAL (CSIC‐UAM) Madrid Spain; ^7^ Istituto Sperimentale Italiano Lazzaro Spallanzani Milano Italy; ^8^ CCMAR Center of Marine Science University of Algarve Faro Portugal; ^9^ RAPID TNO The Hague The Netherlands; ^10^ Department of Pathophysiology and Allergy Research Medical University of Vienna Vienna Austria

**Keywords:** allergenic risk assessment, analytical methods, cross‐reactivity, food allergy, novel food

## Abstract

Food allergies are recognized as a global health concern. In order to protect allergic consumers from severe symptoms, allergenic risk assessment for well‐known foods and foods containing genetically modified ingredients is installed. However, population is steadily growing and there is a rising need to provide adequate protein‐based foods, including novel sources, not yet used for human consumption. In this context safety issues such as a potential increased allergenic risk need to be assessed before marketing novel food sources. Therefore, the established allergenic risk assessment for genetically modified organisms needs to be re‐evaluated for its applicability for risk assessment of novel food proteins. Two different scenarios of allergic sensitization have to be assessed. The first scenario is the presence of already known allergenic structures in novel foods. For this, a comparative assessment can be performed and the range of cross‐reactivity can be explored, while in the second scenario allergic reactions are observed toward so far novel allergenic structures and no reference material is available. This review summarizes the current analytical methods for allergenic risk assessment, highlighting the strengths and limitations of each method and discussing the gaps in this assessment that need to be addressed in the near future.

## 1 Introduction

IgE‐mediated food allergies are regarded as a relevant health concern and affect up to 0.1–3.2% adults and 0.1–5.7% children in Europe.^1^ It is only a limited number of foods that induce an immune response in the majority of predisposed individuals. However, upon contact, allergics can develop rather mild up to severe, even life‐threatening reactions to minute amounts of the causative source.

In order to protect allergic consumers, allergenic risk assessment has been installed. For Europe a list of 14 most commonly allergenic foods and food groups (milk, egg, fish, crustaceans, molluscs, tree nuts, peanut, soy, wheat, lupine, sesame, mustard, celery, and sulfit (for hypersensitivity reasons)) has been established and labeling of these foods is mandatory when used as ingredients.

**Figure 1:**
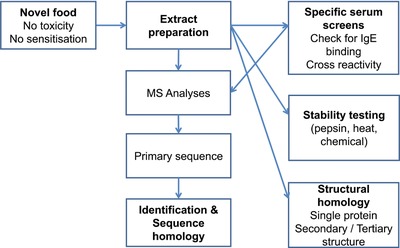
Flow chart on current risk assessment approach for GMO risk assessment adopted for novel foods from EFSA.^2^, ^4^, ^25^ Allergenic risk assessment of novel foods focusing on allergenic cross reactive structures.

Furthermore, for genetically modified organism (GMO)‐derived foods allergenic risk assessment has been established by the European Food Safety Authority (EFSA; **Figure** 1).^2^, ^3^ This risk assessment has been developed within the past two decades and is currently based on a weight of evidence approach. The current GMO risk assessment includes several steps. The newly introduced gene and its product are checked for potential sequence and/or structure similarity to known allergens. In parallel, sera from allergic donors are applied in in vitro assays to investigate whether significant higher IgE binding is observed in the GMO‐product as compared to its isogenic counterpart (specific serum screening). Furthermore, stability of proteins against gastric and duodenal digestion is analyzed, since prolonged stability of proteins may increase the allergenic activity versus labile proteins, which are easily degraded into peptides. Also proteins may vary in their stability when exposed to thermal or chemical treatment; increased resistance may point toward an elevated allergenic risk. Finally, for allergenic risk assessment of GMO foods the comparative compositional analysis of the GM organism versus the isogenic line or appropriate comparators is required.

Since there is a rising need to provide protein sources to feed a steadily growing population, novel sources such as algae and insects are currently explored for safe human consumption. In this context assessing a potential allergenic risk is mandatory before marketing novel food sources.^4^ Prior to assessing the allergenic risk of novel non‐GMO foods or proteins with so far unknown allergenic activity, the valuable existing knowledge about allergenic risk assessment for GMOs needs to be re‐evaluated for its applicability for risk assessment of novel food proteins. Hence, the principles of allergenic risk assessment of GMO will be gauged for their applicability on the assessment of novel foods in this review. Furthermore, the analytical methods applied in this risk assessment will be summarized and recent advances in the methodology highlighted. Also, gaps of knowledge and methodological limitations identified within this assessment will be discussed.

Moreover, the available tools for allergenic risk assessment of potential allergenic food proteins with cross‐reactivity to known allergens may not be applicable for unknown proteins having allergenic potential. Thus, to assess the de novo sensitizing capacity of such novel foods, in vivo testing in animals, or cell culture systems have been proposed and some have shown experimental evidence of principle applicability. However, various challenges of developing and designing animal models and the most important considerations of species and strains, diet, route of administration, relevant controls, and endpoint measures exist, and have been recently reviewed by Bogh et al.^5^ Furthermore, cellular systems such as dendritic cell activation assays have been used to investigate whether the interaction of food and food components with immune cells points toward sensitization or tolerance induction.^6^, ^7^, ^8^, ^9^ Yet, the great majority of these novel methods are not generally available or available at all. Thus, this review focuses on the current knowledge and application of existing and established qualitative and quantitative analytical methods for use in allergenic risk assessment of both food extracts and individual food proteins.

In the following subsections, currently applied methods for purification of allergens from natural food sources as well as their production as recombinant proteins will be summarized. Subsequently, the analytical methods to assess the physicochemical and immunochemical properties are presented, including 2DE coupled with MS, determination of primary, secondary, and tertiary structure of proteins, and IgE‐assays investigating the allergenic activity of proteins. For IgE‐binding assays targeted serum screening, that is applying sera from patients with established specific IgE response to the target food, is essential. In addition, the biological relevance of this specific IgE response can be investigated by basophil activation assays.

In principle two different scenarios of allergic sensitization have to be assessed. The first scenario is the presence of already known or homologous (pan‐) allergenic structures in novel foods (cross‐reactivity). In this case a comparative and targeted assessment can be performed and the range of cross‐reactivity can be explored (Figure 1). In the second scenario allergic reactions are observed toward so far novel allergenic structures and no reference material is yet available (Figure 1). Also, no prospective target serum screening can be performed.^4^ As mentioned above, for the latter scenario well‐defined and predictive cellular assays and animal models are missing.

Hence, this review summarizes the current methods established and applied for in vitro testing, highlighting the advantages and the limitations of each methodology for the allergenic risk assessment of novel food proteins. Both sensitization scenarios are taken into account to identify methods and gaps alike, and to provide information whether the methodology is applicable for single proteins only or for proteins within complex food matrices (**Tables** 1 and 2).

**Table 1:** Methods relevant for allergenic risk assessment

Parameter	Method	Read out	Limitation	Food extract, novel protein, and processed protein	Needs	Relevance for allergenic risk assessment		
						GM foods	Known (pan‐) allergens	Unknown novel proteins
**Amino acid sequence homology**	MS; bioinformatics	Primary sequence; Homology to known sequences	Access to updated allergen / sequence databases	Pure protein, processed protein		✓	✓	–
**Structural Similarity**	CD FTIR, X‐ray based crystallography, and NMR	Secondary and tertiary structure analyses	Shared structure not always linked with allergenicity	Pure protein, processed protein	Scientific evidence of certain structures linked with allergenicity	✓	✓	–
**Aggregation**	SEC; SAXS analysis	Monomers versus oligomers versus polymers	Limited knowledge of aggregation of proteins in processed foods	Pure protein, processed protein	Scientific evidence of link between aggregation & allergenicity	✓	✓	–
**Glycosylation**	LC–MS	Glycosylation: yes/no N‐; O‐glycans; Monosaccharide versus branching	Sensitization with and without clinical relevance	Extracts, pure protein, and processed protein	Scientific evidence of link between glycosylation & allergenicity	✓ (only for certain glycan structures)	✓ (only for certain glycan structures)	–
**Specific serum screening (IgE binding)**	Immunoblot, ELISA, RAST, EAST ISAC ImmunoCAP	Binding to specific IgE	Sensitization with and without clinical relevance; availability of target protein in test systems; and availability of well characterized sera	Extracts, pure protein, and processed protein	Scoring, high throughput; de novo sequencing, when no database available; well characterized patients’ sera	✓	✓	–
**Biological activity testing**	BAT and RBL assay, SPT; Food challenges	Functional IgE testing (cellular assay); Food challenge: Symptoms	Availability of test material; legal and ethical limitations	Extracts, foods, pure proteins, and processed proteins	Availability of well‐defined sera and patients	✓	✓	–
**Resistance to gastric and duodenal digestion**	Standardized digestion assays	% of digested protein; or residual peptide fragments	Complexity of extracts; lack of standardized methods under physiological conditions; not always predicting allergenicity	Extracts, pure protein, and processed protein	Automatization process (high reproducibility, kinetic digestion indexes) guidance to interpretate data. Validation with allergens and nonallergens	✓ Under debate	✓ Under debate	✓ Under debate
**Compar. compos. analysis of GM plant & its appropriate comparator(s)**	Proteomics; LC–MS; and 2DE	Protein amount	Laborious	Extracts, pure protein, and processed protein	Guidance of qualitative and quantitative allergen testing	✓	✓ Only in case of pan allergen assessment	–
**Thermal / chemical stability**	CD FTIR, LC–MS; 2DE	Intact protein versus protein fragments	No single type of structure associated with stability	Extracts, pure protein, and processed protein	Guidance to interpretate data; Validation with allergens and nonallergens			

**Table 2:** Gaps identified in the current risk assessment and recommendations for further research

Methods and tools	Features and limitations	Recommendations for further research
**Allergen databases**	Different databases provide different levels of information; some of them are not regularly updated/curated, and therefore relevant information is missing or available information outdated Inclusion criteria for allergenic proteins vary for individual databases	Linking of existing (allergen) databases; harmonization of inclusion criteria for allergens Experimental studies in B‐ and T‐cell epitopes and implications on cross‐ reactivity Improving predictive algorithms for sensitizing potential of proteins linked with and without clinical relevance
**Analytical methods**	Highly sensitive and advanced methods available for protein characterization Sample preparation especially for complex food extracts is sometimes difficult (lack of harmonized protocols)	Harmonization of method protocols; improvements in sample preparation; generation of scientific evidence of certain structural determinants (glycosylation, aggregation, etc.) linked with increased allergenicity, which is currently lacking
**IgE binding assays**	Well standardized reference assays including reference proteins are missing. In case of novel proteins, no reference material is available; if sIgE is not available, animal derived antibodies can be used	Identification and generation of suitable reference proteins
**Digestion assays**	Different protocols for protein digestion are available; however, harmonized protocols are needed; lack of guidance on how to interpretate data, and lack of reference material; evidence of linking protein stability and de novo sensitization is missing	Development of reference materials and harmonized protocols Performance of harmonized digestion assays in ring trials with reference materials Animal studies on comparative digestion and de novo sensitization
**Food processing techniques**	Knowledge on food processing and its impact on allergenicity is incomplete on a qualitative and quantitative level. Limited knowledge about the most effective methods (combinations), including novel processing techniques	More data on processed food proteins and their allergenicity required; to identify the most important (combination of) processing techniques with an impact on allergenicity
**Food matrix**	Analytical methods are established—but limited data are available showing a link of food matrix components to allergenicity; limited knowledge available about food components and their interaction with allergens	Studies required on food matrix composition and interaction with individual food proteins in model systems; identification of relevant immunomodulating food matrix components
**Biological assays**	Cellular and animal models are established but reliable assays for detection of de novo sensitization are lacking.	Method development to assess protein ligand binding and impact on innate and adaptive immune responses; identification of biomarkers for de novo sensitization

### 1.1 Food Extracts and Purified Allergens

#### 1.1.1 Production of Extracts and Identification of Allergenic Components

For allergenicity assessment of novel foods, preparation of an extract^10^ is the first step, representing all the substances present in the commercial food product, including allergenic and nonallergenic compounds embedded in the food matrix. In fact, it is desirable that the protein extract is made from the processed food as it is consumed, trying to include all possible modifications that could alter the allergenicity. Furthermore, it is important to note that these food extracts may contain (nonproteinaceous) compounds with immunological activity, such as pollen associated lipid molecules in pollen, or secondary metabolites in plants (flavonols and alkaloids).

Usually, these extracts consist of hydrophilic complex mixtures including, proteins, nucleic acids, sugars, and diverse components of the extracellular matrix. Their chemical nature and ratio depend on the origin of the extract. For example, a fruit extract obtained by PBS, contains only 1–5% protein of the dry weight, while the remaining content is mostly sugars. In this case, it is recommended to carry out a selective precipitation for proteins (i.e., with ammonium sulfate or with phosphotungstic acid) to improve their further purification and characterization.^11^

However, for the allergenic characterization of extracts mostly proteins account for the IgE binding activity. In most cases, simple buffers such as PBS or TRIS (pH 7.4) are used, extracting only the readily soluble proteins from the food product.^10^ But proteins display a range of different characteristics, and therefore, the selection of the pH and/or the salt concentration of the extraction buffer is crucial. For example, extraction performed with PBS pH 7.0, usually yields 80–90% of soluble protein content, while, 10–20% are missed.^11^ However, nonspecific lipid transfer proteins (nsLTPs) are soluble at acidic pH (less than 4), while their extraction is reduced down to 40% under neutral pH 7.0 conditions.

Yet, it is also important to determine the optimum conditions to collect allergens from their target food source (e.g. peanut flour versus ground peanut^12^). The food matrix used (e.g. fatty substances) can delay absorption, thus affecting the time interval associated to a reaction, or it may affect the intrinsic allergenic properties of the food.^13^ Hence, it is important to understand how the composition of the food affects the elicitation of a reaction. Therefore, all these factors should be taken into consideration to obtain an extract or complementary extracted fractions that provide a good picture of the novel food. Finally, throughout the extraction procedure endogenous proteoloytic activity must be considered and inactivated if necessary.

#### 1.1.2 Purification of Allergens

In addition to characterizing total food extracts, purification of target proteins is required. Chemical modifications of allergens may happen during the isolation process. For example, seed storage proteins in plants, such as those associated with lipid bodies as oleosins, or deamidation of wheat proteins, may result in structural changes during the purification process. Therefore, it is important to know the nature of the target protein to be purified, and design the least harmful purification protocol.

Purification of allergens/proteins is mostly performed by a series of different extraction and chromatographic techniques.^13^, ^14^ Chromatography is based on the principle of selective retention, which aims to separate the components of a mixture, identifying them, and determining their amounts. There are four main types. Exclusion molecular chromatography (also molecular filtration) separates components by molecular mass. It has low resolution and is usually employed as an initial step in the purification process. Ion exchange chromatography allows separation based on the charge of molecules. Elution is carried out by changing the ionic strength in the mobile phase, either modifying pH or increasing salt concentration. RP chromatography is based on repulsive hydrophobic forces arising from interactions among a relatively polar solvent, a relatively nonpolar compound, and a nonpolar stationary phase. Proteins in the sample are denatured at acidic pH exposing, thus, their hydrophobic regions that are instrumental for separation by polarity. Nonpolar (hydrophobic) regions remain retained longer and are eluted later than polar (hydrophilic) regions. Affinity chromatography is used when specific antibodies are available, and target protein is only available on low concentration in the starting mixture.

Once the allergen has been isolated and purified, the identity and the level of purity need to be assessed. In this regard, amino‐terminal sequencing (increasingly obsolete) and identification by peptide mass fingerprinting and MS are the most frequently used techniques^14^ (see also sub‐section 1.2.2 on protein characterization by proteomics and mass spectrometry). In addition, size exclusion chromatography (SEC) is indicative if comigrating proteins are present in the preparation. Also, further information is needed whether the protein is still in its “native” structure, with respect to aggregation status and/or solubility or potential loss of relevant isoforms etc. (see also Section 1.2).

#### 1.1.3 Recombinant Protein Production and Purification

When purification of allergens from natural sources yield low amounts or is too complex, heterologous expression of allergens represents an alternative. Relatively large amounts (mg–g) of allergens can be produced from heterologous expression systems, potentially with the same biological activity as the natural counterpart. Frequently, the target protein is obtained by cloning and expressing the allergen encoding gene in bacteria (*Escherichia coli*) or yeast (*Saccharomyces cerevisiae* and *Pichia pastoris)*, plants, or other eukaryotic cell systems.^15^, ^16^, ^17^, ^18^

Selection of the optimal expression system depends on the gene involved. For example, *S. cerevisiae* is often preferred for proteins that require significant posttranslational modification while insect or mammal cell lines are used when human‐like splicing of mRNA is needed. Nonetheless, bacterial expression usually has the advantage of easily producing large amounts of target protein as required by X‐ray crystallography or NMR spectroscopy techniques for structure determination. However, since prokaryotes are not equipped with the full enzymatic machinery to accomplish posttranslational modifications, multidomain eukaryotic proteins expressed in bacteria are often nonfunctional. In addition, many proteins become insoluble as inclusion bodies. Consequently, it is very difficult to recover them without harsh denaturants and cumbersome protein refolding procedures need to be performed.^19^

To address these shortcomings, eukaryotic expressions systems have been developed: cells of plants (e.g. tobacco), insects, or mammals are transfected with the target genes and cultured in suspension or even as tissues or whole organisms to produce properly folded proteins. However, in vivo mammalian expression systems show low yield and other limitations (time consuming, toxicity to host cells, etc.). Other expression systems that use unicellular eukaryotes are being developed with the aim of combining high yield production and scalable features of bacteria or yeast proteins with the advanced epigenetic features of plants, insects, or mammals. However, these new systems may present an additional risk of allergenic contamination. Purification of expressed proteins could drag contaminants that act as allergens through cross‐reactivity.^20^ After having successfully established the recombinant production, the respective purification protocol needs to be developed to obtain a highly pure homogenous protein batch. The expression system and the purification process may impact the overall structural integrity and thus allergenic activity. Thus, the recombinant protein should be compared to its natural counterpart, at least when present in an optimized extract, to allow interpretation of its biological activity.

#### 1.1.4 Detection and Characterization of Allergens

##### 1.1.4.1 Food Extracts

To characterize the composition of total food extracts a range of analytical methods can be applied like 2DE followed by MS or LC–MS. For allergenic GMO‐risk assessment comparator extracts are available. However, this is not an option for novel food extracts. Then only batch to batch variation can be assessed and potential marker allergens based on homology screening or MS analysis can be identified. See also the respective sub‐section 1.2.2 on proteomics.

##### 1.1.4.2 Purified Proteins

For purified natural and recombinant proteins the level of purity needs to be determined and additional protein contaminations with and without allergenic activity identified. Also the range of potential isoforms with varying IgE binding capacity needs to be defined as a reference set of proteins.^21^, ^22^ Especially for recombinant proteins lipopolysaccharide (LPS) contamination should be ruled out since this could affect further immunization/sensitization studies. Subsequently, recombinant proteins have to be checked for their physicochemical equivalence to their natural counterpart, that is: do they provide the same posttranslational modifications, such as glycosylation pattern, biological activity, processed/mature protein, correct 3D structure etc.

### 1.2 Allergen Identification: Primary, Secondary, and Tertiary Structure Determination of Allergens (Bioinformatics, LC–MS, CD, and FTIR)

#### 1.2.1 Allergen Databases

It is well known that only a minority of all known proteins identified so far display allergenic activity and these can be assigned to approximately 2% of all known protein families.^23^ Within the last decade efforts have been undertaken to set up a number of allergen databases collecting and curating the existing data of allergen sequences such as the IUIS allergen database (www.allergen.org), AllergenOnline (http: //www.allergenonline.org), Allergome (http://www.allergome.org/), ALLFam (http://www.meduniwien.ac.at/allfam/), and the COMPARE database *(* http://comparedatabase.org/). The IUIS database provides the systematic nomenclature for allergens and is supervised and updated by a specific committee.^24^ Allergenonline provides access to a peer reviewed allergen list and sequence searchable database intended for the identification of proteins that may present a potential risk of allergenic cross‐reactivity. The Allergome database provides a comprehensive, nonpeer‐reviewed collection of information on allergenic proteins, while in the ALLFam database allergens are grouped according to their protein family characteristics. Recently HESI released the COMPARE 2017 Database, a new transparent resource for the identification of protein sequences including known and putative allergens. Current allergenic risk assessment requires the analysis of the primary sequence from a target protein and to assess potential sequence similarity to known allergens.^25^ The alignment based criterion between target protein and already known allergen has been set for 35% sequence identity over a sliding window of 80 amino acid (aa) residues. In addition, several bioinformatics approaches are under development looking for certain motifs or peptides that may contribute to a more precise in silico assessment. It is generally agreed that the quality and reliability of allergen databases are based on their regular updates and continuous curation. With regard to allergenic risk assessment of novel foods, this in silico analysis can only provide information for proteins similar to already known allergens. For any other not yet identified protein this assessment cannot be applied. Whether new algorithms assessing motifs and/or certain peptides related to an increased allergenic activity will improve the current assessment remains to be determined.

#### 1.2.2 Primary Sequence Analysis of Allergens Using MS and Application of Proteomics

The fast evolution of MS and liquid separation techniques highlights promising tools for both, multidetection of known allergens in complex matrices as well as for in‐depth characterization of physico‐chemical properties.^26^ These analyses are also relevant for the quantification of allergens in food products and represent the key information for allergen labeling thereof.

Together with crystallographic and NMR‐based approaches, MS analysis is used for in‐depth allergen characterization.^27^ It includes the study of (a) the primary protein sequence of a target allergen, (b) posttranslational and post food processing modifications, (c) molecular interactions, and (d) structural studies. Primary sequence determination is performed by MS to unambiguously identify allergens. Advanced sequencing methods combine database search and de novo sequencing experiments together with bottom‐up, in which the proteins are digested with trypsin to obtain peptides, middle‐down approach, which produces limited digest (e.g., Glu‐C or Asp‐N), and finally the top‐down approach, which is able to analyze the intact sample without any treatment digestions, which was used for polymorphism studies^28^ evaluating the resistance of allergens against proteolysis^29^ and for sequence homology driven detection of yet unsequenced allergens.^30^

The detection, identification, and localization of allergen modifications require most of the time extensive MS fragmentation capabilities based on different activation techniques and purified proteins. It is also useful for the comparison of native and recombinant proteins. For example, the *N*‑glycome profiling of patatins was performed by the enzymatic release of glycans, followed by chemical derivatization and subsequent MS analyses.^31^ The study of Halim et al. highlighted novel PTMs in major inhalant allergenic proteins.^32^ Recent studies were performed on the detection of advanced glycation end products (AGEs) as well as for the application of related bioinformatic tools.^33^, ^34^ Structural information can be extracted from experiments using ion‐mobility MS and hydrogen deuterium exchange (HDX‐MS) studies.^35^, ^36^ Willison et al. successfully applied HDX‐MS to determine the conformational epitope of Pru du 6.0201, a major allergen from almond.^37^ Intact proteins can be analyzed in the gas phase under a shape similar to that in solution (native MS). Native separation methods such as CE coupled to MS using top down identification strategy is a promising trend of MS.^38^, ^39^ Also online digestion of native proteins may lead to fast epitope mapping.

Proteomics is able to reveal the presence/absence of allergens and thus represents a useful tool to study the composition and the nature of food allergens.^40^, ^41^ Furthermore, the areas of an allergen interacting with IgE antibodies, so‐called B‐cell epitopes, can be determined. Linear epitopes, representing the primary sequence of the allergenic protein are resistant to thermal treatments and thus can be recognized even after reduction processes. In contrast, conformational epitopes are related to secondary and tertiary structure of the protein, are thermo‐labile, and therefore, their identification after protein reduction may not be possible using MS techniques.^42^

The strategies in proteomics to detect food allergens are basically two: one is based on a gel approach while the other represents a gel‐free approach. The gel‐based approach includes 1DE and 2DE followed by immunoblotting and MS to identify the protein spots. The gel‐free approach is based on HPLC–MS/MS and subsequent IgE binding assay of the trypsinized proteome. Bioinformatic analysis is needed for the prediction of specific immunoreactive epitopes in both approaches. Previously, several studies reported the successful detection of allergens in foods using a 2D‐immunoblotting experimental design, in beer,^41^ beef,^42^ milk,^43^, ^44^ rice,^45^ fish,^46^ and so on. As mentioned above glycans can trigger IgE‐based immune reactions. In meat, mostly alpha‐Gal is responsible for allergic reactions. The work of Apostolovic and colleagues identified novel alpha‐Gal‐containing proteins by 2DE.^42^ Fish is one of the most frequent causes of IgE‐mediated food allergy and is a good example for the application of proteomics in food allergen detection and modulation. Jamaluddin et al. published a study about parvalbumins in the longtail tuna (*Thunnus tonnggol*). In addition to the parvalbumin identification two new major thermolabile allergens were identified as creatine kinase and enolase.^47^ Another interesting approach was performed by Rodrigues et al., who modulated parvalbumin expression in fish muscle. Gel‐based proteomics was used to follow the modulation of parvalbumin expression in the European seabass fed with specifically designed low‐allergen diets to be used for farmed fish production.^48^

The possibility to detect allergens even in trace amounts in complex food matrices is challenging. It is well‐known that also little quantities, not indicated in the food label, could represent a serious burden for highly sensitive allergic patients. Recently, huge progress in allergen quantification has been made by the use of Multiple Reaction Monitoring MS (MRM–MS). Specific peptide signatures derived from allergens can be quantified by Selected Reaction Monitoring acquisition (SRM) or MRM and the use of internal peptide standards.^49^ A recent tendency in the field of MS based quantitative analyses relies on the use of High Resolution full scan MS acquisition (HR‐MS). This acquisition mode renders possible comprehensive analyses in a non‐targeted approach. Korte et al. applied this method for multi‐allergen detection screening with the additional option for retrospective detection.^50^ Koeberl and colleagues developed a method for the quantification, using MRM.^51^ They authored an interesting paper where they reported pro and cons of both, immunological and MS methods and highlighted the importance of crucial selection of each antigen of signature peptides and related transitions. Moreover, Houston and colleagues^52^ succeeded in the detection of the concentration of ten allergens in commercial soybean varieties using a label free proteomics approach. By using MRM approach, they were able to measure antigens in the range between 0.5 and 0.7 *μ*g mg^–1^. They used BSA as an internal standard in order to reduce the technical variance up to 7%. Another example is the fast detection of fish allergen parvalbumin (one of the major allergens in fish) described by Carrera and colleagues who proposed a selected MS/MS ion monitoring (SMIM) in a linear ion trap (LIT) mass spectrometer, which can be performed in less than 2 h.^53^

#### 1.2.3 Secondary Structure Determination of Proteins: CD and FTIR, and X‐Ray and NMR

*Basic Principles of CD Spectroscopy*: Circular dichroism (CD) spectroscopy has become an invaluable research technique for gaining information about protein structure, dynamics, and interactions with both, other proteins and ligands, respectively.^54^ Two basic types of information can be obtained from CD spectra:

- Secondary structure composition (% of alpha‐helix, beta‐sheet, beta‐turn, random coil, etc.) from the peptide bond region (far‐UV region, or “far‐UV CD”)
- Tertiary structure fingerprint from the CD spectra of aromatic aa residues (near‐UV region or “near‐UV CD”).

#### 1.2.4 Secondary Structure Calculation from Far‐UV CD Spectra

Different types of secondary structures give rise to characteristic CD spectra in far‐UV (below 250 nm), which differ in their peak positions and intensities.^55^, ^56^ Various empirical methods have been developed for analyzing protein CD spectra for quantitative estimation of the secondary structure content. However, reference database sets of proteins, with CD spectra matching to secondary structure components derived from X‐ray structures, provide the key resource for this task. Thus, the most important variable that contributes to the quality of the CD spectra analyses for the prediction of protein secondary structure is the reference database that is used.

#### 1.2.5 Tertiary Structure Fingerprint from Near‐UV CD Spectra Analysis

The spectra in the region 260–320 nm arise from the aromatic aas. The actual shape and magnitude of the near UV CD spectrum of a protein will depend on the number of each type of aromatic aa present, their mobility, the nature of their environment, and their spatial disposition in the protein. The near UV CD spectrum of a protein can be used to compare, for example, wild type and mutant forms of proteins, but may also point to food processing‐induced changes in proteins.^57^ It can also provide important evidence for the existence of ‘‘molten globule’’ states in proteins, which are characterized by practical absence of near UV CD signals, due to high mobility of aromatic side chains. These “molten globule” states are often present as intermediates of thermal denaturation of globular proteins.^58^

Both, far and near CD spectra have been useful in monitoring thermal stability of food allergens, i.e. thermal stability of Cor a 14, the 2S albumin from hazelnut.^59^ The effects of high‐pressure/temperature treatment and pulsed electric field treatment on peanut Ara h 2 and 6 and apple Mal d 3 and 1b were examined by CD spectroscopy^60^ and the results showed that novel processing technologies had little effect on the allergen structure. Both, far and near CD spectra were also useful in assessing the effects of high intensity ultrasound on the structure of beta‐lactoglobulin, major allergen of cow's milk, confirming moderate effects of the processing on protein's fold and stability.^57^

#### 1.2.6 Fourier Transform IR Spectroscopy in Structural Characterization of Proteins

Fourier transform IR (FTIR) spectroscopy is another nondestructive technique for structural characterization of proteins and polypeptides. The IR spectral data of proteins are interpreted in terms of the vibrations of a structural repeat. The repeat units in proteins give rise to nine characteristic IR absorption bands (amides A, B and I–VII). Amide I bands (1700–1600 cm^−1^) are the most prominent vibrational bands of the protein backbone, and they relate to protein secondary structural components.^61^ The method has been widely applied to the secondary structure and structural dynamics analyses, assessment of conformational changes, and stability studies of food proteins. FTIR measurement of secondary structure helps to assess protein aggregation and stability, thus making this technique of strategic importance in the food proteomic field. Examples of applications of FTIR spectroscopy in the study of structural features of food proteins critical of nutritional and technological performance are discussed in several review articles.^62^ It has been shown that increasing the temperature clearly affects the shape of the amide I band in FTIR and indicates formation of beta‐structures. These data show that denaturation of peanut Ara h 1 leads to a more structured conformation of the protein and explains the observation that Ara h 1 aggregates upon heating, a process that is known to coincide with the formation of antiparallel beta‐sheet structures.^63^ Aggregation of allergens at elevated temperatures also prevents recording of far‐UV CD spectra. Therefore, FTIR is the method of choice for confirming structural features of aggregated and poorly soluble proteins.

#### 1.2.7 Tertiary Structure Determination Methods

*X‐Ray and NMR in Solution*: Protein tertiary structure (3D) has been investigated using a number of spectroscopic approaches such as X‐ray crystallography, NMR, and MS. X‐ray crystallography is the most powerful method to obtain information about the tertiary structure of proteins at the atomic level. In the recent past improvement of computational technologies and the development of new and powerful computer programs together with the enormous increment in the number of protein structures deposited in the Protein Data Bank (PDB), facilitated the resolution of new structures.^64^ X‐ray crystallography provides detailed, atomic‐level information about a protein structure. However, it is not always possible to obtain crystals of the required quality for such analysis. NMR offers a reasonable alternative to X‐ray crystallography for low‐molecular‐weight proteins.^65^

Our understanding of allergens has greatly improved due to large sets of proteins being crystallized and/or resolved by NMR and their structures deposited in the PDB.^66^ The first enzyme structure to be determined was lysozyme, which happens to be the chicken allergen, Gal d 4.^67^

In addition to the information obtained on the structure of individual allergens, structure analyses of allergens in complexes with their natural ligands were also performed.^35^, ^68^, ^69^ These X‐ray structures of molecular complexes allow to identify the atoms involved in the molecular interactions between allergens and molecules of our immune system. These complexes include peptides presented by MHC class II molecules,^70^ cytokines bound to their receptors,^71^ allergen–antibody complexes,^72^, ^73^ and innate immune receptors with their ligands.^74^ Determination of the 3D structure of allergens has revealed the existence of new structural groups of proteins. Alt a 1, the major allergen from *Alternaria alternata*, has a unique beta‐barrel structure that forms a ‘‘butterfly‐like’’ dimer and is exclusively found in fungi.^75^ The cockroach allergen, Bla g 1, has an alpha‐helical structure thus far only found in insects.^27^ For some of important food allergens, such as Ara h 2, X‐ray structure was solved in a form of hybrid expressed with mannose‐binding protein (MBP).^76^

#### 1.2.8 Computational Methods in Prediction of Tertiary Structure

Due to the advances in computational methods and the steadily growing number of protein data bank (PDB) entries, predicting protein structures, based only on aa sequence becomes a reasonably fast, simple, and reliable method. The methods of computational prediction rely on modeling based on structural similarity of aa sequences between target protein and a homologous protein of known 3D structure. There are webservers available, such as the IntFOLD webserver, which provides a simple unified interface that aims to make complex protein modeling data more accessible to life scientists.^77^ Molecular modeling of three tegumental allergen‐like proteins from *Schistosoma mansoni* showed that despite similarities in the domain organization, there are differences in the structures of the three proteins.^78^ Three‐dimensional models of the major vicilin allergens from peanut (Ara h 1), lentil (Len c 1), and pea (Pis s 1), were built by homology‐based modelling from the X‐ray coordinates of the structurally closely related soybean beta‐conglycinin.^79^ In addition, before the structure of Ara h 2 was solved by X‐ray crystallography, its 3D structure was modeled from Ara h 6 (a homologous peanut allergen), which was solved by NMR.^80^

### 1.3 Specific Serum Screening–IgE Binding Assays

Food allergic reactions are built up in two phases. After the first encounter with a given food source the predisposed individual mounts a Th2 dominant immune response and produces allergen specific IgE antibodies, called sensitization phase. After repeated exposure to the same allergenic source the IgE antibodies present on the surface of mast cells recognize the specific allergen, cross‐link, and activate mast cells to release their biogenic amines (e.g. histamine) and immunoactive substances,^81^ which then cause local or systemic symptoms, termed effector phase. It is generally accepted that allergic sensitization comprises the presence of serum derived IgE antibodies and does not always coincide with allergic symptoms.

Specific serum screening, that is using serum from an individual sensitized to a given food source, can be performed by different in vitro assays. Depending on the assay a qualitative or quantitative evaluation can be obtained. All in vitro IgE tests require a reference protein (purified and well characterized natural or recombinant allergen) or extract (standardized reference extracts) and anti‐IgE antibodies highly specific for this isotype.^82^ To assess the overall lgE binding activity of an extract, Western blots provide information about IgE binding proteins on a qualitative level (e.g., size and presence of different proteins). Primary separation of proteins can be done one‐ or two‐dimensionally. ELISA assays allow an overall quantification of IgE binding capacity to a target extract. Also diagnostic assays used for clinical routine can be applied to assess the IgE binding activity of a novel food source or purified proteins thereof. These routine tests are available in different formats. In principle, the extract or single proteins are immobilized to a solid phase capturing specific antibodies from serum samples. Subsequently, bound IgE is detected and quantified by labeled anti‐IgE antibody detection reagent, e.g. enzyme conjugated monoclonal anti‐IgE antibodies. Some solid phase assays can adsorb high amounts of single proteins or extracts, whereas others use a microarray format, spotting a high number of minute amounts of individual highly pure allergens simultaneously. Depending on the assay the required amount of serum varies between 30 μL up to 120 μL. Appropriate controls and reference protein batches need to be tested in parallel (purified natural or recombinant allergens, standardized extracts, etc.). While some tests provide absolute quantification of specific IgE, others, such as the microarray approaches, allow semiquantitative assessment of IgE antibody concentrations.

IgE binding and cross‐linking by allergens can also be analyzed using functional in vitro, ex vivo, and in vivo testing. Cellular in vitro or ex vivo assays such as basophil activation test (BAT) can be performed to confirm functional IgE binding. Upon stimulation with allergens or extracts cellular activation is measured either by mediator release or upregulation of cellular surface molecules. In this assay allergens and extracts in different buffers can be tested. In vivo, skin prick testing and oral food challenges can be performed. Skin prick testing (SPT), verifies specific mast cell degranulation. This test is applied in clinical routine diagnosis, using total extracts or the target food source on human skin. After 15 min a wheal and flare reaction can be observed as a positive test result. For clinical diagnosis purified recombinant allergens are not applied in SPTs due to regulatory issues. However, SPTs demonstrate allergic sensitization, but the correlation to clinical symptoms is limited. Oral food challenges (OFCs), including the double blind placebo controlled food challenge (DBPCFC), which is considered the gold standard of food allergy diagnosis, is also an option to be included in allergenic risk assessment. However, these tests are time and cost consuming and are restricted to specialized medical centers. Since uniform standardized procedures are lacking, the test outcomes are difficult to compare. Therefore, harmonized test protocols and validated challenge materials are needed.^82^

If a novel food is introduced into the diet, it may induce allergic reactions in already sensitized individuals due to the presence of cross‐reactive proteins.^25^ Alternatively, it may contain novel allergens, and thus induce allergic reactions. While cross‐reactions will appear rather quickly after first contacts, de novo sensitizations will be recognized later. For allergenic risk assessment cross‐reactivity can be assessed using sera from already sensitized donors, while for de novo sensitization this is not possible.

For allergenic risk assessment the availability of well‐defined patients’ sera is a limiting factor for IgE based in vitro assays. Especially for novel foods where no previous allergic sensitizations have been reported these assays can only be performed to check for potential cross‐reactivity based on sensitization to well‐known allergens such as profilins, Bet v 1‐homologues, nsLTPs, etc. Regarding the risk of de novo sensitization to previously unknown allergens, there is a need to develop new assays, such as cellular tests following sensitization, or identifying physicochemical parameters of proteins highly relevant for allergic sensitization. Also animal derived specific antibodies can be produced and applied in immunological tests (ELISA, Western blots). For allergenic risk assessment of novel foods, post market monitoring could help to investigate the potential risks of novel foods.

### 1.4 Pepsin Resistance and In Vitro Digestibility Tests

In order to understand the allergic response, it is important to study the impact of digestion on food allergens, with regard to their structural integrity to induce T‐cell differentiation and IgE‐mediated activation of effector cells after gastrointestinal transit. In 1996, Astwood et al. developed a protocol for digestion studies using simulated gastric fluid containing the enzyme pepsin in order to discriminate between nonallergenic and allergenic proteins.^83^

However, further studies are hesitant on the use of pepsin resistance as a criterion for predicting allergenicity, as it has been shown that many allergenic proteins are not more resistant than nonallergenic ones.^84^, ^85^ The apparent stability of a protein may vary depending on the experimental conditions used (pH, pepsin–protein ratio, purity, and chemical environment). In addition, food processing and components of the food matrix can drastically affect the digestion of allergens.^86^ Therefore, more complex models than pepsinolysis, generally multiphasic, considering relevant factors in the physiological digestion, have been developed.^87^, ^88^, ^89^, ^90^ Nevertheless, there is a lack of correlation between digestibility and allergenicity, probably due to the different conditions of digestion assays. In order to consolidate conditions for simulated digestion, a harmonized digestion model has been recently proposed.^91^ However, this model was not yet used to measure the difference between the digestibility of allergenic and nonallergenic proteins.

In addition to different experimental protocols, different interpretation of the results, could explain the disparity of results. Thus, for example, Prs‐1 avocado allergen is fully digested in simulated gastric fluid in less than 1 min; however, the resulting peptides retain their IgE‐binding epitopes and exhibit a reactivity similar to that of the intact protein both in vitro and in vivo.^92^ This highlights the importance of using high‐resolution analytical techniques combined with more sensitive detection methods, as well as a reference set of allergens in tests.^93^

Although the resistance to digestion is regarded as one of the common properties of food allergens, digestibility does not provide sufficient information for allergenicity prediction, so the study of the impact of the digestion process, at a molecular level, is still essential to analyze the remaining protein or polypeptide structures that could play a role in sensitizing or symptom triggering.

### 1.5 Selected Examples for Well‐Known Allergens and Novel (Potentially Allergenic) Proteins

When assessing the allergenicity of a novel food or food protein, we can generally describe two major cases: (1) The novel food or food protein likely contains pan‐allergenic and thus potentially sensitizing and/or cross‐reactive structures. (2) By contrast, no comparative information about potential sensitizing or cross‐reactive structures is available, therefore the allergenic potential of the novel food or protein is completely unknown. Both scenarios will be further discussed.

According to structural, biochemical, and functional characteristics, the so far identified allergenic proteins are found within a fairly limited number, i.e. approximately 2%, of protein families compared to all known protein families.^23^ Of the plant‐derived foods, the most frequent allergens are among the prolamin and cupin superfamilies, the profilins, and within the pathogenesis‐related PR‐10 protein family. Examples are allergenic globulins and albumins of seed storage proteins, ns‐LTP, and proteins related to the major birch pollen allergen Bet v 1.^23^ Among the animal‐derived foods, allergens are frequently found within the protein families of tropomyosins, EF‐hand proteins, lipocalins, caseins, and serum albumins.^23^, ^94^

Thus, when assessing allergenicity of novel foods or proteins that likely contain or constitute proteins within the above mentioned protein families with known allergenic potential, the methods described in this paper can be used to gain knowledge about the similarity to known allergens, the inherent stability to allow substantial interaction with the immune system, and the presentation of functional, and thus potential allergenic epitopes. Accordingly, allergenic risk assessment has been based on methods to perform compositional analysis, to elucidate the primary protein sequence and secondary and tertiary structural elements, to investigate resistance against simulated gastric digestion, and to clarify in a targeted approach of the IgE binding and cross‐linking properties of the novel food protein(s).

One such example where novel allergenic structures were identified in novel plant foods is the study of Gubesch and colleagues^95^ about three exotic vegetables from Africa, Asia, and South America that have not been commonly consumed in the EU, namely water spinach (*Ipomea aquatica*; Convolvulaceae), hyacinth bean (*Lablab purpureus*, syn.*Dolichos lablab*; Fabaceae), and Ethiopian eggplant (*Solanum gilo*; Solanaceae). The authors applied a three‐step procedure that included (1) the identification of pan‐allergenic structures, (2) the verification of in vitro IgE‐binding capacity of these structures, and (3) in vivo verification of functional mediator release using SPT, and OFC with the novel vegetables upon positive SPT. Pan‐allergenic structures were identified by immunoblot analyis with cross‐reactive animal antibodies immunized against PR‐10 proteins, profilins, LTPs, and allergenic legume storage proteins. The targeted in vitro screening of IgE binding to the vegetable total protein extracts was accomplished with serum IgE from subjects allergic to Fabaceae and Solanaceae foods, Mediterranean food allergics with LTP sensitization, and subjects having multiple pollen‐associated food allergies. SPT and OFC were done in subjects with pollen (grass, birch, and mugwort) allergy but who had not been exposed to the three novel vegetables before. In summary, profilin and LTP were first identified by animal antibodies in all vegetables, and a Bet v 1 homologue in hyacinth bean. Second, IgE‐binding to LTP, profilin, and a Bet v 1 homologue was proven by immunoblot analysis and EAST (Enzyme‐Allergosorbent‐Test). Third, positive SPT and OFC results were observed for all vegetables in the pollen‐allergic patients. Thus, in vivo testing proved the potential of the novel vegetables to elicit clinical allergy and verified the in vitro findings.

Another striking example of a novel allergenic animal food is the insect Yellow mealworm (*Tenebrio molitor*) protein^96^ where pan‐allergenic structures were identified and their IgE‐binding and cross‐linking properties demonstrated. The authors used approaches as advised by EFSA for allergenicity assessment. Making use of LC–MS/MS, a large number of proteins in Yellow mealworm extracts (YMP) was identified, among which tropomyosin (TM), and arginine kinase (AK) are proteins already identified as major or important allergens in e.g. crustaceans. In silico analysis of the identified aa sequences, using Allermatch^TM^ against the UniProt database, and applying the criteria of >35% identity within an 80 aa sliding window, suggested several hits for known allergens of mites and insects. It is worth noting that mites, insects, and crustaceans are taxonomically closely related and belong to the arthropods. As, e.g. TM and AK are considered pan‐allergenic structures in arthropods, individuals with allergies to other arthropods such as crustacean or house dust mite (HDM) might be at risk when consuming YMP. Consequently, the authors demonstrated in vitro IgE binding and functional basophil activation of YMP in patients with crustacean and HDM allergy, and AK and TM were identified as major cross‐reactive allergens in YMP. Finally, simulated gastric fluid digestion proved a moderate stability of YMP. The demonstrated realistic possibility that HDM and Crustacean allergic patients may react to food containing YMP was further verified in an in vivo follow‐up study.^97^ In DBPCFC, 13 of 15 patients allergic to shrimp reacted positive to mealworm. A comparison of individual eliciting doses of four patients who also had a shrimp challenge indicated that the eliciting doses as well severity were in the same range for Yellow mealworm and shrimp. In conclusion, the in vivo testing proved the potential of the novel insect protein to elicit clinical allergy and verified the in vitro findings.

Both examples about the identification of known allergenic structures lead to the conclusion of a realistic possibility of clinical cross‐reactivity in subjects with preexisting allergy to these structures. Obviously, this approach fully depends on targeted screening based on the potential allergenicity of known allergenic structures.

By contrast, any novel protein without structural, biochemical, or functional relationship cannot be assessed for potential allergenicity with the methods described in this paper. Other approaches are needed that can investigate the sensitizing potential, such as dendritic cell activation or mouse models, to discriminate between allergens and nonallergens.^5^, ^98^

Moreover, it needs to be emphasized that even the identification of known allergenic or pan‐allergenic structures does not necessarily prove allergenicity. A well‐known example is coiled‐coil‐superhelical TM, an invertebrate pan‐allergen,^99^ but a nonallergen in vertebrate meat such as from beef, pork, or chicken.^100^ The identities between nonallergenic vertebrate and allergenic invertebrate tropomyosins are below ≈60%. By contrast, the identities of nsLTPs from peach and kiwi may be comparably low but IgE cross‐reactivity is retained.^101^ Further, the substitution of only 12 aa of the allergenic TM, Pen a 1 from Brown shrimp toward vertebrateTMs, while retaining the secondary structure as assessed by CD spectroscopy leads to 90–98% reduction of allergenicity as measured in functional specific mediator release from humanized rat basophilic leukemia cells.^102^ These 12 aa resemble only 4% difference in aa identity.

A similar example can be found within the PR‐10 protein family. The major birch pollen allergen, Bet v 1, is considered the primary sensitizer in birch‐associated soybean allergy. Allergic reactions to soybean in birch pollen allergic subjects are mediated by the homologous soybean allergen, Gly m 4.^103^ For IgE binding, the conserved fold is required.^104^ By contrast, Norcoclaurine synthase (NCS) from meadow rue, which is structurally homologous to Bet v 1 does not bind Bet v 1^105^ or Gly m 4 specific IgE.^104^ By grafting a very limited number of critical aa residues of IgE‐binding surface areas of Bet v 1 or Gly m 4 onto NCS, IgE binding is induced.

In summary, the mere structural relationship or degree of aa identity does not seem to explain the differences in allergenicity. Thus, aa identity and structural homology alone are weak predictors in allergenicity risk assessment of novel foods, and additional tests are needed to assess their allergenic potential.

## 2 Summary and Conclusions

There is an increasing need for novel food sources to feed a growing population while ensuring safe human consumption. Therefore, a potentially increased allergenic risk of novel foods has to be assessed. With the use of up to date analytical techniques, primary, secondary, and tertiary structures of proteins can be investigated down to a molecular level. For the allergenic activity assays are available, using either IgE antibodies derived from patients’ sera or antibody sera raised in animals. Also, cellular assays provide evidence of functional allergenic activity of food proteins. In addition, digestion assays have been used to investigate the stability of proteins as another indicator of potential allergenicity, and food processing and food matrix interaction with allergenic proteins is considered to impact on allergenicity, too. In parallel, databases collating sequences and their physicochemical and immunological features have evolved, facilitating the search for homologous proteins and peptides.

For the current risk assessment strategies it has to be clearly stated that not a single method is available to predict allergenicity between a novel allergenic versus nonallergenic molecule, both at the sites of elicitation and sensitization. Therefore, a weight of evidence approach is needed. Furthermore, the established and currently applied methods are based on a homology assessment to already identified allergens. Thus, these methods are of limited applicability for novel proteins lacking homology to already identified proteins. Independently, even for the well‐established methods used in allergenicity risk assessment of homologous proteins, we have identified limitations or gaps of knowledge (Table 2) which should be addressed in the future. For example, various protocols exist for the performance of IgE‐binding assays, digestion assays, and biological assays. Certainly, there is a need for harmonization of protocols or at least guidance in calibrating the assays with appropriate reference proteins that present low and high allergenicity, respectively. The identification and availability of such reference proteins may help comparing data sets obtained by the various assays. Inclusion criteria for allergenic proteins in databases should be harmonized for better comparability and sound data mining. Experiment‐based knowledge about allergenic B‐ and T‐cell epitopes or mimotopes is still limited. Availability of such knowledge in allergen databases may allow improving algorithms for prediction of a sensitizing potential of proteins independent of basic structural homology. Collaborative ring trials should determine the robustness of individual techniques, ideally performed in a blinded setting. More data on how food processing, and food matrix interaction such as ligand binding, modify the allergenicity of proteins, are required if such knowledge should be used in predictive models.

For the allergenic risk assessment of novel foods or food proteins without homology to known allergenic structures, additional cellular (e.g. dendritic cell) assays and animal models need to be developed and validated, ideally using known allergenic and known nonallergenic reference proteins of nonhomologous sources. Within the COST action Improving Allergy Risk Assessment Strategy for New Food Proteins (ImpARAS), a partnership including academia, industry, stakeholders, and regulators, the gaps of the current risk assessment using established techniques are addressed, and the practical implications of existing and future test procedures and parameters will be discussed. Further, the COST action ImpARAS will work toward novel strategies to better predict safe consumption of novel foods based on the findings about the underlying immunological mechanisms of an allergic response, especially addressing the event of sensitization.

**Abbreviations**
aa: amino acid
AGEs: advanced glycation end products
AK: arginine kinase
CD: circular dichroism
DBPCFC: double blind placebo controlled food challenge
EAST: Enzyme‐Allergosorbent‐Test
EF‐hand protein: proteins with characteristic helix‐loop‐helix domain
EFSA: Eureopan Food Safety Authority
FTIR: Fourier transform infrared
HDM: House dust mite
HDX‐MS: Hydrogen deuterium exchange
nsLTP: nonspecific lipid transfer protein
OFC: oral food challenge
PALM: pollen associated lipid mediators
PDB: Protein data bank
PR‐10: pathogenesis related protein family 10
PTA: phosphotungstic acid
PTMs: post translational modifications
sIgE: specific immunoglobulin E
SPT: skin prick test
SRM: selected reaction monitoring
TM: Tropomyosin

## Conflict of Interest Statement

T.H. had consultant arrangements with “Institut fuer Produktqualitaet,” Berlin, Germany, and with Monsanto Company, St. Louis, Missouri, USA. He received fees from “Die Akademie Fresenius,” Dortmund, Germany, for speaking and supporting education, and received reimbursement for attending and supporting symposia and education from European Academy of Allergology and Clinical Immunology, Zurich, Switzerland; Deutscher Allergie und Asthmabund eV,Moenchengladbach, Germany; International Life Science Institute/Health and Environmental Sciences Institute, Brussels, Belgium, and Washington, DC; International Association of Food Protection, Des Moines,Illinois, USA; AOAC International, Rockville, Maryland, USA; SAG Suederelbe Projektgesellschaft AG & Co.KG, Hamburg, Germany; Canadian Food Inspection Agency, Ottawa, Ontario, Canada; Deutsche Gesellschaft fuer experimentelle und klinische Pharmakologie und Toxikologie eV, Mainz, Germany; DFG German Research Foundation, Bonn, Germany; and BLE German “Bundesanstalt fuer Landwirtschaft und Ernaehrung,” Bonn, Germany—all outside the submitted work.

KHS received reimbursement for attending and supporting symposia and education from European Academy of Allergology and Clinical Immunology, Zurich, Switzerland; is a member of the EFSA ad hoc working group on allergenic risk assessment of GMOs; all outside the submitted work.

All other authors (A.D.P., K.V., Pa.R., Pe.R., E.M., T.C.V., and G.M.) declare no conflict of interest.
